# Unveiling synapse pathology in spinal bulbar muscular atrophy by genome-wide transcriptome analysis of purified motor neurons derived from disease specific iPSCs

**DOI:** 10.1186/s13041-020-0561-1

**Published:** 2020-02-19

**Authors:** Kazunari Onodera, Daisuke Shimojo, Yasuharu Ishihara, Masato Yano, Fuyuki Miya, Haruhiko Banno, Naoko Kuzumaki, Takuji Ito, Rina Okada, Bruno de Araújo Herculano, Manabu Ohyama, Mari Yoshida, Tatsuhiko Tsunoda, Masahisa Katsuno, Manabu Doyu, Gen Sobue, Hideyuki Okano, Yohei Okada

**Affiliations:** 1 Department of Neurology, Aichi Medical University School of Medicine, 1-1 Yazakokarimata, Nagakute, Aichi 480-1195 Japan; 2 Department of Neurology, Nagoya University Graduate School of Medicine, Nagoya, 466-8550 Japan; 3 Department of Physiology, Keio University School of Medicine, Tokyo, 160-8582 Japan; 4 Division of Neurobiology and Anatomy, Graduate School of Medical and Dental Sciences, Niigata University, Niigata, 951-8510 Japan; 5 Department of Medical Science Mathematics, Medical Research Institute, Tokyo Medical and Dental University, Tokyo, 113-8510 Japan; 6 Department of Biological Sciences, Graduate School of Science, The University of Tokyo, Tokyo, 113-0033 Japan; 7 Laboratory for Medical Science Mathematics, RIKEN Center for Integrative Medical Sciences, Yokohama, 230-0045 Japan; 8 Department of Pharmacology, Hoshi University School of Pharmacy and Pharmaceutical Sciences, Tokyo, 142-8501 Japan; 9 Department of Dermatology, Keio University School of Medicine, Tokyo, 160-8582 Japan; 10 Department of Neuropathology, Institute for Medical Science of Aging, Aichi Medical University, Nagakute, Aichi 480-1195 Japan; 11 Research Division of Dementia and Neurodegenerative Disease, Nagoya University Graduate School of Medicine, Nagoya, 466-8550 Japan

**Keywords:** Spinal bulbar muscular atrophy, Induced pluripotent stem cells, iPSC-derived motor neurons, RNA sequencing, Gene set enrichment analysis, Synapse, Neurotransmitter, Neuromuscular junctions, Epigenetics, Endoplasmic reticulum

## Abstract

Spinal bulbar muscular atrophy (SBMA) is an adult-onset, slowly progressive motor neuron disease caused by abnormal CAG repeat expansion in the androgen receptor (AR) gene. Although ligand (testosterone)-dependent mutant AR aggregation has been shown to play important roles in motor neuronal degeneration by the analyses of transgenic mice models and in vitro cell culture models, the underlying disease mechanisms remain to be fully elucidated because of the discrepancy between model mice and SBMA patients. Thus, novel human disease models that recapitulate SBMA patients’ pathology more accurately are required for more precise pathophysiological analysis and the development of novel therapeutics. Here, we established disease specific iPSCs from four SBMA patients, and differentiated them into spinal motor neurons. To investigate motor neuron specific pathology, we purified iPSC-derived motor neurons using flow cytometry and cell sorting based on the motor neuron specific reporter, *HB9*^*e438*^*::Venus*, and proceeded to the genome-wide transcriptome analysis by RNA sequences. The results revealed the involvement of the pathology associated with synapses, epigenetics, and endoplasmic reticulum (ER) in SBMA. Notably, we demonstrated the involvement of the neuromuscular synapse via significant upregulation of Synaptotagmin, R-Spondin2 (RSPO2), and WNT ligands in motor neurons derived from SBMA patients, which are known to be associated with neuromuscular junction (NMJ) formation and acetylcholine receptor (AChR) clustering. These aberrant gene expression in neuromuscular synapses might represent a novel therapeutic target for SBMA.

## Introduction

Spinal bulbar muscular atrophy (SBMA) is an adult-onset slowly progressive lower motor neuron (MN) disease caused by abnormal CAG repeat expansion in the androgen receptor (AR) gene. SBMA is characterized by weakness and atrophy of limbs and bulbar muscles caused by the degeneration of spinal and bulbar MNs [1–3]. Through analysis using transgenic mice models harboring mutant AR with expanded polyglutamine tract (AR-97Q) and in vitro cell culture models, mutant AR has been shown to form nuclear aggregation in a ligand (testosterone)-dependent manner, causing neuronal cell death through transcriptional dysregulation, impaired axonal transport, and other mechanisms [4, 5]. However, mechanisms underlying neuronal degeneration in SBMA are not yet fully elucidated, as SBMA model mice exhibit several discrepancies with human SBMA patients. For instance, the number of CAG repeat required for the onset of the disease is 38 or more in patients [6, 7], but more than 90 in transgenic mice models [8]. Moreover, the skeletal muscle degeneration is more prominent in model mice than in human patients [8]. From the aspect of therapeutics, although leuprorelin acetate, a luteinizing hormone-releasing hormone (LHRH) analogue, dramatically improved symptoms of AR-97Q mice [9], it could not ameliorate the symptoms of human SBMA patients, as had been observed in model mice, and induced an antianabolic action of skeletal muscle as an adverse effect [10, 11]. Therefore, a novel human disease model that more accurately recapitulates SBMA patients’ pathology has been expected for more precise pathophysiological analysis and the development of novel therapeutics.

Interestingly, recent analyses have shown that phenotypes and MN pathology of transgenic mice models were unexpectedly rescued by muscle-specific silencing of mutant AR, suggesting the involvement of muscle pathology in motor neuronal degeneration [12, 13]. In addition, impaired transmission of neuromuscular synapses was observed in SBMA mice models [14]. These results strongly indicate muscular pathology, which causes neurodegeneration in SBMA. However, the molecular pathology underlying neuromuscular interactions has not been sufficiently investigated, due to the lack of appropriate disease models.

Disease specific human induced pluripotent stem cells (hiPSCs) provide valuable disease models for neurodegenerative disorders [15, 16]. They are able to give rise to otherwise unavailable neural cells, which more accurately model patients’ pathology and provide us with new platforms for pathophysiological analysis and drug discovery [17]. For instance, α-synuclein accumulation observed in PARK2 iPSC-derived dopaminergic neurons well corresponded to the pathological findings of the Lewy body pathologies observed in autopsied brain specimens of the same patient [18]. In addition, MNs derived from sporadic amyotrophic lateral sclerosis (ALS)-iPSCs exhibited a time course of neuronal degeneration consistent with the clinical course observed in corresponding ALS patients [19].

On the other hand, iPSC-derived disease models still have issues to overcome, including time and labor required for the establishment and differentiation of iPSCs, heterogeneities of differentiated neural cells, and large variations among iPSC clones. In particular, the phenotypes or molecular changes may be masked by variations of the differentiation efficiency among iPSC clones, indicating the necessity of a method to purify target cells. To overcome these issues, we previously established rapid, efficient, and simple MN differentiation system from hiPSCs, in which MN progenitors could be obtained within two weeks with the induction efficiency of HB9^+^-MNs of approximately 40–50%. Moreover, MNs could be enriched by flow cytometry and cell sorting with MN specific *HB9*^*e438*^*::Venus* reporter lentivirus [20].

In this study, we established iPSCs from four SBMA patients, differentiated them into MNs, and investigated molecular pathology behind the motor neuronal degeneration in SBMA. We focused on common molecular changes among multiple iPSC clones derived from different patients. Differentiated MNs were purified using the lentiviral reporter system to overcome clonal variations of iPSCs, and to elucidate MN specific pathology. Furthermore, genome-wide transcriptome analyses by RNA sequences revealed the involvement of the key molecular pathology associated with synapses, epigenetics, and endoplasmic reticulum (ER) in SBMA. Pathology associated with neuromuscular synapses such as Synaptotagmin proteins, RSPO2, and WNT ligands were particularly highlighted, which may serve as novel therapeutic targets for SBMA.

## Results

### Establishment of SBMA disease specific iPSCs

**Fig. 1:**
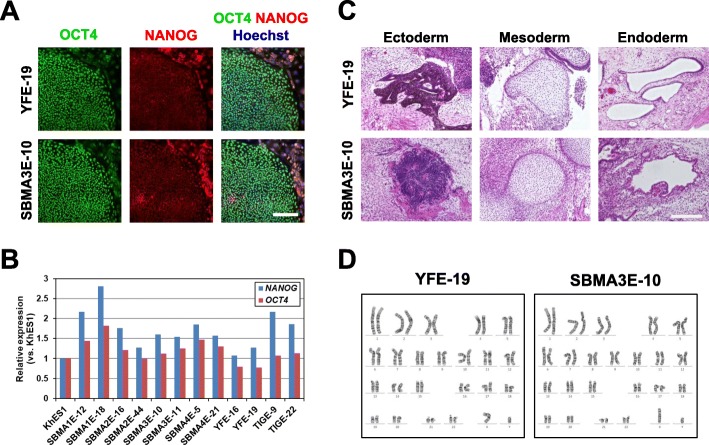
Establishment of iPSCs from SBMA patients and controls. **a** Immunocytochemical analysis of YFE-19 and SBMA3E-10 iPSCs for pluripotent markers OCT4 (green) and NANOG (red). Scale bar, 100 μm. The established iPSC clones were positive for OCT4 and NANOG. **b** Quantitative RT-PCR analysis of the expression of pluripotent makers *OCT4* and *NANOG* in the established iPSC clones. Data are normalized to *β-ACTIN* and presented as the relative expressions to KhES1 (hESC). All established iPSC clones expressed *OCT4* and *NANOG*. **c** Hematoxylin and eosin staining of teratomas derived from YFE-19 and SBMA3E-10 iPSCs. Scale bar, 200 μm. The established iPSC clones were able to differentiate into 3 germ layers. **d** Karyotype analysis of YFE-19 and SBMA3E-10 iPSCs via G-banding analysis. The established iPSC clones showed normal karyotypes, 46, XY. See also Additional file 1: Figures S1 and S2, Additional file 2

We first established iPSCs from skin fibroblasts isolated from four SBMA patients (SBMA1, 2, 3, and 4), and three healthy age-matched control adults (TIG114, YF, KN) (Table 1), by introducing *OCT4*, *SOX2*, *KLF4*, *L-MYC*, *LIN28,* and *shTP53* via episomal vectors. Three of the control iPSC clones, TIGE-9, YFE-16, and EKN3, have already been described previously [20, 21] (Table 1). All the SBMA and control iPSC clones showed characteristic human embryonic stem cell-like morphology and expressed pluripotent stem cell markers, Oct4 and Nanog, as revealed by immunocytochemical analysis (Fig. 1a and Additional file 1: Figure S1). The expressions of *OCT4* and *NANOG* were also confirmed by quantitative RT-PCR, which showed comparable expression to KhES1 (human embryonic stem cells) [22] in all the clones examined (Fig. 1b). We also examined teratoma forming capacity, which showed differentiation potentials into all three germ layers (Fig. 1c and Additional file 1: Figure S1), and normal karyotypes by G-banding (Fig. 1d and Additional file 2: Figure S2). These results suggest that we have established iPSC clones with the quality necessary for pathophysiological analysis.

**Table 1:** Characterization of iPSCs

	Name	Race	Gender	Age	CAG repeat	iPSC clones
SBMA	SBMA1	Japanese	male	42	52	SBMA1E-12, 18
	SBMA2	Japanese	male	46	47	SBMA2E-16, 44
	SBMA3	Japanese	male	33	50	SBMA3E-10, 11
	SBMA4	Japanese	male	38	49	SBMA4E-5, 21
Control	TIG114	Japanese	male	36	24	TIGE-9^a^, 22
	YF	Japanese	male	24	21	YFE-16^a^, 19
	KN	Japanese	male	39	20	EKN3^b^

All SBMA patients were not treated with leuprorelin at the time of skin biopsy

^a^ [20], ^b^ [21]

### Stability of CAG repeat numbers during reprogramming

**Fig. 2:**
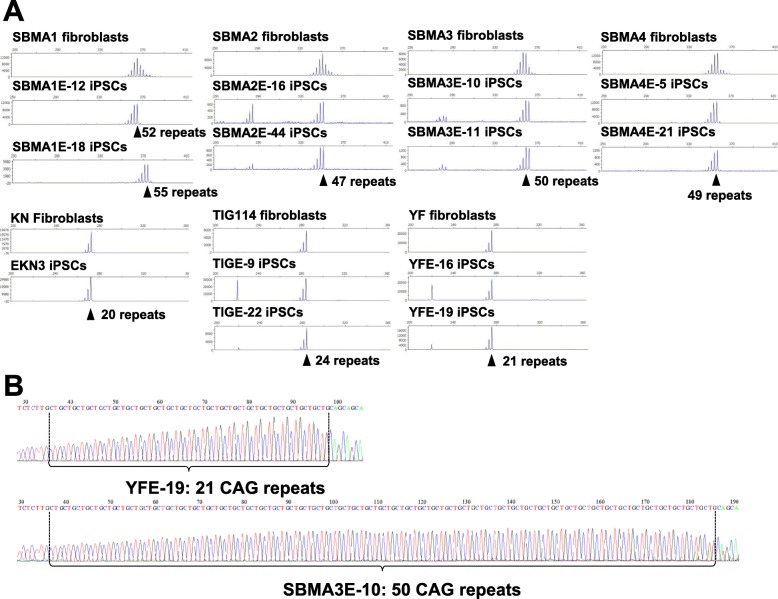
Stability of CAG repeats in the AR gene during reprogramming. **a** CAG repeat determination by fragment analysis. CAG repeat number in the AR gene was stable during reprogramming in the established iPSC clones, except for SBMA1E-18, which showed 55 CAG repeats. **b** Direct sequence analysis of CAG repeats in genomic DNA from YFE-19 and SBMA3E-10 iPSCs showed 21 and 50 CAG repeats, respectively. Repeat sequencing was read from the 3′ side

To determine the stability of expanded CAG repeat during reprogramming, we next examined the length of the CAG repeat in the AR gene by fragment analysis. As expected, the length of the CAG repeat in each iPSC clone was stable during reprogramming, except for SBMA1E-18, whose number of CAG repeat increased from 52 to 55 (Fig. 2a). These results were confirmed by direct Sanger sequencing, as shown in Fig. 2b (representative images of YFE-19 and SBMA3E-10 are shown) and Table 1. These results suggest that the CAG repeat number was maintained during the reprogramming process in SBMA.

### Differentiation of SBMA disease specific iPSCs into MNs

**Fig. 3:**
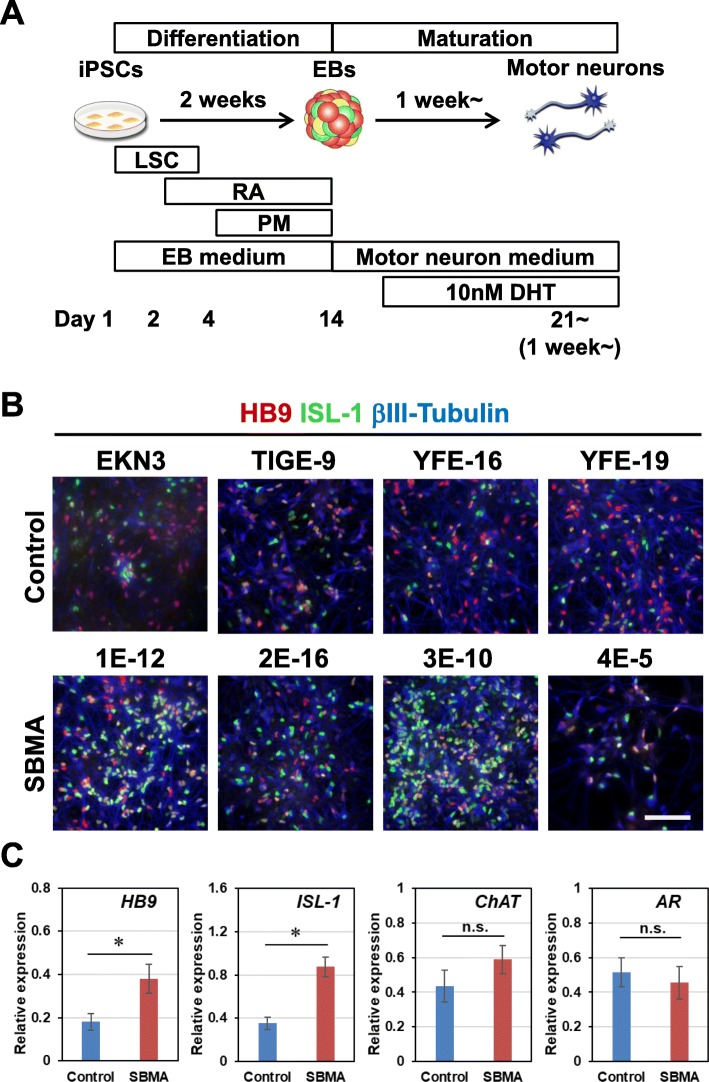
Differentiation of SBMA disease specific iPSCs into motor neurons. **a** Schematic presentation of the culture protocol for the differentiation of iPSCs. LSC, LDN-193189 (L), SB4315342 (S), CHIR99021 (C); RA, retinoic acid; PM, purmorphamine. **b** Immunocytochemical analysis of HB9, ISL-1, and βIII-Tubulin after 1 week of monolayer differentiation with 10 nM DHT. Scale bar, 100 μm. **c** Quantitative RT-PCR analysis of *HB9*, *ISL-1*, *ChAT*, and *AR* expressions at 4 weeks (*n* = 4, mean ± SEM). *HB9* and *ISL-1* expressions were higher in SBMA than controls in the presence of 10 nM DHT (*p < 0.05; Student’s t-test)

To confirm the differentiation potentials of established iPSC clones, control and SBMA iPSC clones were induced to differentiate into MNs by the previously reported method [20]. After 2 weeks of embryoid body (EB) formation, which contains MN progenitors, the EBs were dissociated into single cells and adherently differentiated and maturated on poly-L-ornithine- and laminin-coated cover glasses or plates for 1–4 weeks (maturation culture) (Fig. 3a). In one week, we confirmed differentiation into HB9^+^ and ISL-1^+^ MNs from all SBMA and control iPSC clones by immunocytochemistry (Fig. 3b). According to quantitative RT-PCR analysis, the expressions of *HB9* and *ISL-1* in SBMA iPSC-derived MNs were higher than those in control iPSC-derived MNs, while mature MN markers *ChAT* and *AR* did not show significant differences at 4 weeks of maturation culture (Fig. 3c). These results suggest that all the established clones have the potential to sufficiently differentiate into MNs, and exhibit some clonal variations for the differentiation propensity among hiPSC clones.

### Purification of *HB9*^*e438*^*::Venus* positive MNs by flow cytometry and cell sorting

**Fig. 4:**
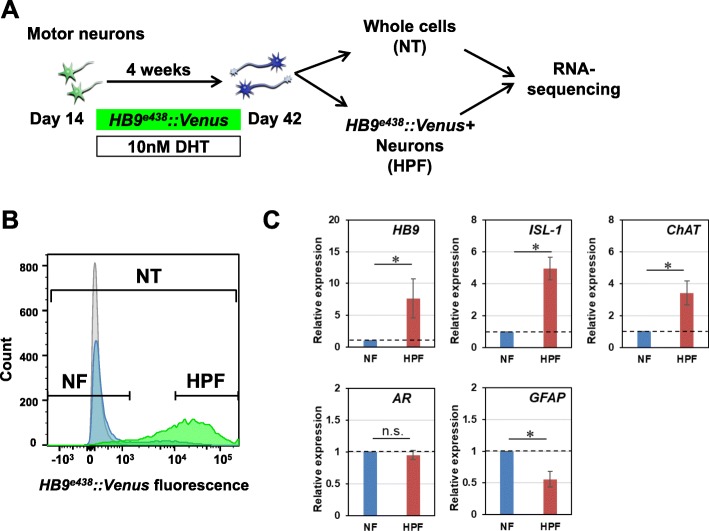
Purification of *HB9*^*e438*^*::Venus* positive MNs by flow cytometry. **a** Schematic presentation of the culture protocol for flow cytometry and cell sorting. **b** Schematic histogram for determining a negative fraction (NF) and high-positive fraction (HPF). NF consists of uninfected cells (gray). HPF consists of top 2/3 fraction of *HB9*^*e438*^*::Venus*^+^ cells (green). NT consists of unpurified cells. Blue, background lentiviral infected cells. **c** Quantitative RT-PCR analysis of *HB9*, *ISL-1*, *ChAT*, *AR,* and *GFAP* expressions at 4 weeks. Data are normalized to *β-ACTIN* and presented as the relative expressions to controls (*n* = 4, means ± SEM). Significant increases in the expressions of *HB9*, *ISL-1*, and *ChAT* are observed in the HPF. A significant decrease in the expression of *GFAP* is observed in the HPF (*p < 0.05; Student’s t-test)

For the transcriptome analysis using purified/enriched populations of MNs, MNs were infected with the *HB9*^*e438*^*::Venus* reporter lentivirus four days after the initiation of the maturation culture, and were cultured for a further four weeks in the presence of 10 nM dihydrotestosterone (DHT), which is a ligand of AR, to detect ligand-dependent pathology of SBMA. The cells were then detached and dissociated into single cells, and purified by flow cytometry and cell sorting based on *HB9*^*e438*^*::Venus* fluorescence (Fig. 4a). The cells were divided into 3 fractions, a negative fraction (NF: gated by the fluorescence intensity of uninfected controls, gray peak), positive fraction (PF: green peak), where the top 2/3 of PF was determined as high-positive fractions (HPF; purified MN fraction), and others (Fig. 4b), following previously reported protocol with some modifications [20]. We also examined the expression of Venus fluorescent protein from a reporter lentivirus containing the same vector backbone, but without the *HB9*^*e438*^ enhancer element (background reporter lentivirus; blue peak in Fig. 4b) and found that the fluorescence observed in the HPF of *HB9*^*e438*^*::Venus* reporter-transduced cells overlapped minimally with the fluorescence generated by the background reporter. We further confirmed the expression of the MN markers, *HB9*, *ISL-1* and *ChAT*, and astrocyte maker, *GFAP,* by quantitative RT-PCR using NF and HPF. We observed significant increases in the expression of MN markers and a decrease in the astrocyte maker in HPF. The expression levels of *HB9*, *ISL-1,* and *ChAT* in HPF were 7.7 ± 3.1-folds, 5.0 ± 0.7-folds, and 3.4 ± 0.7-folds higher than those in NF (*n* = 4, *p < 0.05*) (Fig. 4c), respectively, suggesting HPF was highly purified MN fraction. Although the expressions of *HB9* and *ISL-1* were higher in SBMA iPSC-derived MNs compared with those of control iPSC-derived MNs before purification, which indicates clonal variation of the differentiation efficiencies (Fig. 3c), they were equally expressed in all the clones after purification, suggesting that the clonal variation was corrected by the purification of MNs by flow cytometry and cell sorting based on *HB9*^*e438*^*::Venus*. The significant decrease in the expression of *GFAP* indicated the elimination of non-MNs/astrocytes from MN cultures by purification (0.56 ± 0.12-folds, *n* = 4, *p* < 0.05). AR expression was comparable in the NF and the HPF, consistent with the finding that AR expression was not specific to MNs.

### Differentially expressed genes between SBMA- and control-MNs by RNA sequencing

**Fig. 5:**
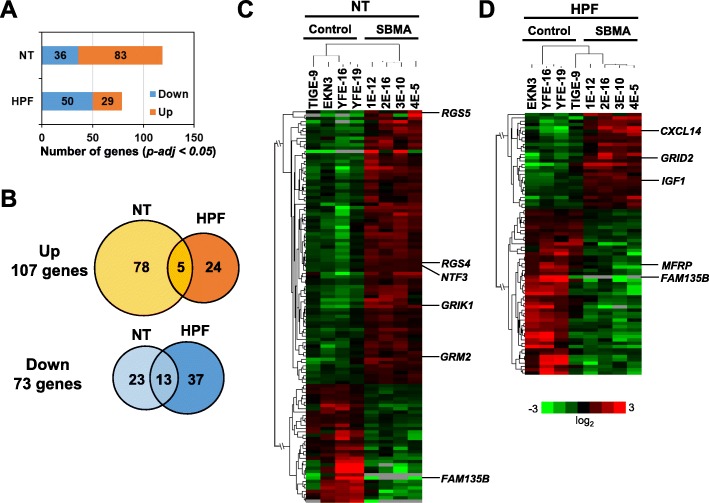
Identification of differentially expressed genes (DEGs) by RNA sequencing analysis. **a** Number of DEGs in the RNA-seq data, adjusted *p-value* (*p-adj*) *< 0.05*. The number of genes with *p-adj < 0.05* in NT was 119, and 79 genes in HPF from a total of 58,825 annotated genes. **b** The Venn diagrams of up-regulated or down-regulated genes in SBMA (*p-adj < 0.05*). Up-regulation of 107 genes and down-regulation of 73 genes are shown. **c** The heatmap of the hierarchical clustering analysis based on the DEGs with *p-adj < 0.05* in NT. Red represents higher expression and green indicates lower expression. For the list of the genes, see also Additional file 3: Table S1. **d** The heatmap of hierarchical clustering analysis based on the DEGs with *p-adj < 0.05* in HPF. Red represents higher expression and green indicates lower expression. For the list of the genes, see also Additional file 3: Table S2

**Fig. 6:**
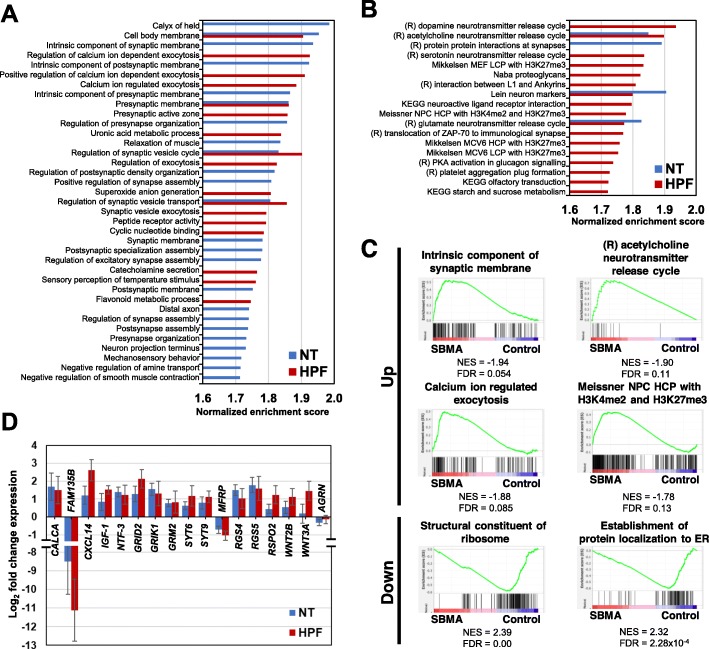
Enrichment of synapse-related pathology by GSEA. **a** Gene ontology (GO) enrichment analysis. Up-regulated gene sets in SBMA with FDR < 0.2 are shown. Blue indicates enriched in NT, and red in HPF. For negatively enriched GO terms, see also Tables S4 and S5, Additional file 3. **b** Pathways significantly enriched in SBMA with FDR < 0.2 are shown. Blue indicates enriched in NT, and red in HPF. (R), Reactome is a curated database of pathways and reactions in human biology. For negatively enriched pathway terms, see also Tables S6 and S7, Additional file 3. **c** Representative enrichment plots. Synapse, neurotransmitter, exocytosis, and epigenetics associated gene sets were involved in enrichment of SBMA. Ribosome and ER were negatively enriched in SBMA. The green curves show the enrichment score and reflect the degree to each black line, which shows a position of a gene in the ranked list of genes. NES, normalized enrichment score. FDR, false discovery rate. ER, endoplasmic reticulum. **d** The focused gene expression from DEGs and GSEA. The vertical axis represented relative log2 fold change (SBMA / Control) in the gene expression (*n* = 4, mean ± SEM)

To investigate disease-associated genes in SBMA-MNs, total RNA was extracted from both unpurified MNs (NT) and purified MN fraction (HPF) from MNs derived from 4 SBMA-iPSCs and 4 control-iPSCs, and processed for RNA sequencing analysis. As a result, 119 genes showed adjusted *p-values* less than 0.05 in NT, and 79 genes in HPF (Fig. 5a). In total, of these differentially expressed genes (DEGs) in NT and HPF, 107 genes were upregulated and 73 genes were downregulated in SBMA-MNs (Fig. 5b). The heatmap of the DEGs showed that the majority of DEGs in NT were upregulated genes in SBMA-MNs, while the majority of DEGs in HPF were downregulated genes (Fig. 5c, d). All the ranked genes were shown in Additional file 3: Tables S1 and S2. Among these DEGs, *CXC motif chemokine ligand 14* (*CXCL14*), *Insulin-like growth factor-1* (*IGF-1*), *Neurotrophin-3* (*NTF3,* NT-3), *Glutamate ionotropic receptor delta type subunit 2* (*GRID2*), *Glutamate ionotropic receptor kainate type subunit 1* (*GRIK1*), *Glutamate metabotropic receptor 2* (*GRM2*), *Regulator of G protein signaling 4* (*RGS4*) and *Regulator of G protein signaling 5* (*RGS5*) are markedly upregulated, and *Family with sequence similarity 135 member B* (*FAM135B*) and *Membrane-type frizzled-related protein* (*MFRP*) are prominently downregulated, as shown in Fig. 5c, d, and Fig. 6d. As dysregulation of these DEGs are also previously shown to be associated with neurodegeneration, they could also be involved in the pathology of SBMA.

### Involvement of synapse pathology in SBMA shown by GSEA

To investigate important pathways and signals involved in SBMA pathology, we next performed gene set enrichment analysis (GSEA) [23]. The dataset has 58,825 native features. After collapsing features into gene symbols, there are 57,238 genes remaining. In the gene ontology (GO) analysis, gene set size filters (minimum = 15, maximum = 500) resulted in filtering out 4652 / 9996 gene sets. The remaining 5344 gene sets were used in the analysis. In the pathway analysis, gene set size filters resulted in filtering out 1406 / 5501 gene sets. The remaining 4095 gene sets were used in the analysis. The numbers of gene sets either up- or down-regulated in SBMA is listed in Additional file 3: Table S3.

GO analysis revealed that the gene sets that were enriched in SBMA-MNs included gene sets related to synapse in both NT and HPF; those related to synaptic vesicles, calcium ions, and exocytosis in HPF (Fig. 6a). Pathway analysis revealed that gene sets related to neurotransmitters were enriched in SBMA-MNs in both NT and HPF (Fig. 6b). Among these gene sets, acetylcholine neurotransmitter release cycle could be closely associated with synaptic pathology in MNs, and all gene sets were associated with the synaptic function and development of NMJs. In addition, the gene sets associated with histone methylation, including H3K4me2 and H3K27me3, were also enriched in SBMA in HPF, and located at higher ranks, suggesting that epigenetics was also involved in the pathogenesis of SBMA, as previously reported [24]. Representative enrichment plots which were significantly enriched in SBMA with regards to synapse, neurotransmitter, exocytosis, and epigenetics are shown in Fig. 6c (upper 4 panels). The members of each gene set (black lines) appear close to SBMA (red zone). This distribution indicates significant enrichment in SBMA.

As for negatively enriched gene sets in SBMA-MNs in GO analysis, chromosome and ribosome related gene sets were occasionally detected in both NT and HPF. Furthermore, in HPF, in addition to the gene sets of mRNA catabolic processes and ribosomes, the gene sets of endoplasmic reticulum (ER) are positioned at higher ranks (Additional file 3: Tables S4 and S5), suggesting that there is decreased protein synthesis in SBMA-MNs and the involvement of ER in SBMA pathology (Additional file 3: Tables S4-S7). Representative enrichment plots which were significantly enriched in control-MNs, including ribosome and ER are shown in Fig. 6c (lower 2 panels). The members of the gene set (black lines) appear close to the control (blue zone).

Finally, we investigated genes that are particularly elevated in SBMA-MNs, among gene sets detected by GO and pathway analyses, using a FDR q-value < 0.2, fold change ≧ 2, and *p < 0.05*. As a result, 96 genes were extracted (Additional file 3: Table S8), including *Calcitonin-related polypeptide α* (*CALCA*), which has previously been reported to be involved in SBMA pathogenesis [25], *GRID2*, *GRIK1*, *R-spondin 2* (*RSPO2*), *Synaptotagmin6* (*SYT6*), *Synaptotagmin9* (*SYT9*), *Wnt family member 2B* (*WNT2B*) and *Wnt family member 3A* (*WNT3A*), most of which are involved in the synaptic functions in NMJs independent of agrin (Fig. 6d). Agrin (*AGRN*), which induces acetylcholine receptor (AChR) clustering at NMJs, did not show significant alteration (Fig. 6d), suggesting that neuromuscular pathology, independent of agrin, plays crucial roles in the pathogenesis of SBMA (Fig. 6d).

## Discussion

### Stability of CAG repeat numbers in SBMA disease specific iPSCs

Some of the triplet repeat diseases exhibit trinucleotide repeat instability, which results in the increase of repeat numbers, earlier disease onset, and increased severity of the disease in successive generations, known as anticipation. In SBMA, such an instability of the CAG repeat has not been observed in somatic and germ cells [26], and the anticipation is rare [7]. These observations are consistent with our results, in which CAG repeat expansions in the *AR* gene were relatively stable during reprogramming to establish iPSCs. Similar results were obtained in the previously established SBMA disease specific iPSCs, in which CAG repeat numbers were unaltered during reprogramming, long-term maintenance of iPSCs, and differentiation [27, 28], except for one report that showed variations in the repeat number, possibly due to the mosaicism of parental fibroblasts [29].

However, the consistency between the trinucleotide repeat instability in patients’ tissues and those in iPSCs is still not clear in other triplet repeat diseases, such as Huntington disease (HD), caused by CAG repeat expansion in the *Huntingtin* (*HTT*) gene, and myotonic dystrophy (DM1), caused by CTG repeat expansion in the 3′ UTR of the *DMPK* gene, both of which exhibit somatic instability of the repeats, and significant anticipation [30]. Although the augmentation of the CAG repeat expansion was not observed during reprograming into iPSCs, the long-term maintenance of iPSCs, and the neural differentiation in HD disease-specific iPSCs [31, 32], all 41 iPSC clones established from DM1 patients’ fibroblasts showed different CTG repeat length as expected [33]. Thus, trinucleotide repeat instability during reprogramming does not completely correspond to that observed in patients’ tissues, and the details remain yet to be elucidated.

### Identification of cytokines and neurotrophic factors involved in motor neuron degeneration

Since mutant AR is known to translocate into the nucleus of MNs in a ligand (testosterone)-dependent manner, causing transcriptional dysregulation and MN degeneration in SBMA [5, 8], iPSC-derived MNs were treated with 10 nM DHT for 4 weeks to detect ligand-dependent pathogenesis. A previous report has shown that SBMA iPSC-derived MNs demonstrated relatively mild cellular phenotypes without mutant AR aggregations for up to 30 days of MN differentiation culture in the presence of DHT [34]. Since our SBMA iPSC-derived MNs were also cultured for up to 4 weeks in this study, they may have recapitulated similar molecular changes to those observed in the above-mentioned pathological stages without mutant AR aggregations.

Through RNA sequence analysis of four SBMA-MNs and four control-MNs, we identified 79 and 119 DEGs in purified (HPF) and unpurified (NT) MNs, respectively. Only 18 genes were commonly identified, suggesting the purification of MN enabled the detection of MN-specific pathology (Fig. 5b). More importantly, several molecules which have been previously reported to be associated with SBMA pathology, such as *CALCA* and *FAM135B*, were also identified [25, 34], indicating reproducibility of the previous findings in our iPSC-derived SBMA model. Among DEGs, several cytokines and neurotrophic factors were upregulated in SBMA-MNs, including *CXCL14*, *IGF-1,* and *NTF3*. *CXCL14* is known to be upregulated in mice exhibiting similar phenotypes to ALS [35], suggesting that it may also be involved in MN degeneration in SBMA. *IGF-1* is shown to suppress apoptosis of a mutant AR expressing MN cell line (MN-1) by phosphorylating AR via Akt, which inhibits ligand-dependent translocation of the mutant AR into the nucleus [36]. IGF-1 and Pituitary adenylyl cyclase activating polypeptide (PACAP) rescued the reduction of depolarizing current and electro-physiologically improved MN-1 cells that express mutant AR (AR100Q) [37]. Moreover, muscle specific overexpression of *IGF-1* rescued the phenotypes of SBMA model mice, which include mutant AR aggregations through Akt-dependent phosphorylation of the mutant AR [38]. Thus, the upregulation of *IGF-1* in SBMA-MNs could be attributable to the compensatory negative feedback of rescuing the pathology of SBMA. *NTF3* (NT-3) is primarily known to be expressed and involved in the development of proprioceptive neurons in dorsal root ganglia [39, 40]. NT-3 also promotes survival and regeneration of MNs [41, 42], and formation and function of NMJs, including neuromuscular transmission [43, 44]. Thus, the upregulation of *NTF3* in SBMA-MNs could induce increased synapse density or could be the result of compensatory negative feedback, associated with neuronal or neuromuscular synapse degeneration.

### GSEA revealed core gene sets involved in the pathogenesis of SBMA

According to GSEA, upregulation of (1) synapse-, (2) neurotransmitter-, (3) calcium-related exocytosis-, and (4) epigenetics-related gene sets, and downregulation of (5) ER-related gene sets were identified as involved in the pathogenesis of SBMA-MNs. Notably, in purified MNs (HPF), gene sets related to activated synapse function or those related to epigenetics were more significantly enriched in SBMA-MNs.

Regarding epigenetics, the gene sets of high-CpG-density promoters (HCP) bearing the methylation mark at K4 and K27 (H3K4me2 and H3K27me3) were enriched in the HPF of the SBMA-MNs. Although abnormal histone modification has never been reported, DNA methyltransferase1 is known to be strongly expressed in SBMA model mice and patients’ spinal cords, which could be rescued by its inhibitor through the suppression of *Hes5* [24]. As the role of epigenetics is also reported in the etiology of ALS [45], epigenetics may play important roles in the neurodegeneration present in SBMA.

As negatively enriched gene sets, ER-related gene sets are identified. ER plays crucial roles in the unfolded protein response (UPR), which is the ER protein quality control pathway. The deficiency of the UPR mediator, CHOP, was shown to activate macro-autophagy, which is a lysosomal protein quality control pathway and which accentuated phenotypes of SBMA model mice [46]. Moreover, ER-associated calcium homeostasis was shown to be disturbed in cultured embryonic MNs using SBMA model mice [47]. Therefore, down-regulation of ER-related gene sets suggests dysregulation of UPR in the SBMA-MNs.

### Pathology in neuromuscular synapses were highlighted to cause neurodegeneration in SBMA

Most highlighted differences between the SBMA- and control-MNs were within synapse-related gene sets. Recently, the skeletal muscle specific overexpression of *IGF-1*, or the silencing of mutant AR, was shown to rescue the phenotypes of SBMA model mice, suggesting the involvement of skeletal muscles in the neurodegeneration in SBMA [12, 13, 38]. Moreover, by analyzing NMJs in the SBMA model mice, pathological fragmentation of NMJs and abnormal synaptophysin distribution, as well as defects in the neuromuscular synaptic transmission, were demonstrated, which is consistent with the previously reported insufficient synaptic functions in SBMA [14, 48]. Interestingly, our transcriptome analysis of iPSC-MNs revealed upregulation of synapse-related gene sets in the SBMA-MNs, including activation of intracellular signaling via calcium ions, formation of synaptic vesicles, and the release of neurotransmitters such as acetylcholine. Similarly, analysis of DEGs, synapse-related genes were abundantly enriched and were found to be upregulated in the SBMA-MNs (Figs. 5c, d, and 6d). For instance, glutamate receptors *GRID2*, *GRIK1,* and *GRM2* could be involved in glutamate toxicity in MNs, a phenomenon reported to occur in ALS. Upregulation of these genes in the SBMA-MNs suggests the involvement of similar glutamate toxicity in SBMA. Another example is that RGS proteins, including *RGS4* and *RGS5*, negatively regulate G protein-coupled receptor (GPCR) signaling, and work in coordination to regulate key aspects of neurotransmitter release, synaptic transmission, and synaptic plasticity at neuronal synapses [49]. Increased expressions of *RGS4* and *RGS5* inactivate GPCR signaling, which could induce neurodegeneration [50]. Moreover, the synaptotagmin family, including *SYT6* and *SYT9*, plays important roles in calcium-dependent exocytosis of synaptic vesicles, and *SYT9* has been shown to be involved in the pathology of ALS [51].

As for the neuromuscular synapse formation, *RSPO2* is highly expressed in MNs and directly binds to its receptor, leucine-rich repeat-containing G-protein coupled receptor 5 (Lgr5) at the neuromuscular synapse. *RSPO2* not only activates canonical Wnt pathways, but also promotes AChR clustering via muscle-specific tyrosine kinase (MuSK) and low-density lipoprotein receptor-related protein 4 (Lrp4) [52, 53]. Similarly, some of the Wnt ligands (Wnt4, Wnt7a, Wnt7b, Wnt9a, Wnt9b, Wnt10b, Wnt11, and Wnt16) also promote AChR clustering independent of Agrin [54–56], but *WNT3A* negatively regulate NMJ formation [57]. Thus, increased expression of *RSPO2*, and Wnt ligands in SBMA-MNs, could induce abnormal neuromuscular synapse formation, or could be caused by compensatory negative feedback of pathological synaptic degeneration.

In this study, SBMA disease-specific iPSCs provided a valuable disease model that enabled elucidation of previously unknown pathologies underlying neurodegeneration. Comprehensive transcriptome analysis using purified MNs revealed MN-specific and patient-specific pathologies that have never been investigated in detail. Notably, this analysis further highlighted synaptic pathologies in SBMA. In contrast to most of the previous studies which focused on muscular pathology causing non-cell autonomous neurodegeneration and NMJ dysfunctions, we demonstrated that neuromuscular synapses and synaptic functions of NMJs could be affected by MN pathology in SBMA, and also identified alteration of gene expressions in MNs, such as Synaptotagmin, RSPO2, and WNT ligands. Through these analysis, identification of novel therapeutic targets focusing on neuromuscular synapses is expected.

## Materials and methods

### Isolation of human skin fibroblasts and generation of iPSCs

Human dermal fibroblasts (HDFs) were obtained from 4 Japanese SBMA patients and 3 controls: a 36-year-old Japanese male from the Japanese Collection of Research Bioresources (JCRB) Cell Bank (TIG114), a 24-year-old Japanese male (YF), and a 39-year-old Japanese male (KN). HDFs were cultured in DMEM (Sigma-Aldrich, USA), 10% fetal bovine serum (FBS; Sigma-Aldrich, USA), 2 mM L-glutamine, and 1% penicillin/streptomycin. Then, 6 × 10^5^ HDFs were transfected with 1 μg of each of the following: pCXLE-hOCT3/4-shp53 (*OCT4* and *shTP53*), pCXLE-hSK (*SOX2* and *KLF4*), and pCXLE-hUL (*L-MYC* and *LIN28*; a gift from Dr. Yamanaka). Plasmid transfection was performed using the Neon transfection system (Thermo Fisher Scientific, USA). After 6 days, the cells were harvested and plated on mitomycin-C-treated SNL murine fibroblast feeder cells in 0.1% gelatin-coated tissue culture dishes in human fibroblast medium. On the next day, the medium was changed to standard human embryonic stem cell (hESC) medium containing DMEM/F-12 (Wako, Japan), 20% knockout serum replacement (KSR) (Thermo Fisher Scientific, USA), 2 mM L-glutamine, 1% non-essential amino acids (NEAA) (Sigma- Aldrich, USA), 0.1 mM 2-mercaptoethanol (2-ME) (Sigma- Aldrich, USA), 0.5% penicillin/streptomycin, and 4 ng/mL recombinant human fibroblast growth factor-2 (FGF-2) (Peprotech, USA). When the colonies had grown to a sufficiently large size, they were picked and expanded in the same way as hESCs and hiPSCs. The properties of the established iPSC clones were evaluated, as described previously (Fig. 1, Additional files 1 and 2: Figure S1 and Figure S2). Control iPSC clones, which were TIGE-9, TIGE-22, YFE-16, YFE-19, and EKN3, and the SBMA iPSC clones, which were SBMA1E-12, SBMA1E-18, SBMA2E-16, SBMA2E-44, SBMA3E-10, SBMA3E-11, SBMA4E-5, and SBMA4E-21, were established. The control iPSC clones (TIGE-9, YE-16, and EKN3) were previously reported [20, 21]. For the RNA sequence analysis, control iPSC clones (EKN3, TIGE-9, YFE-16 and YFE-19) and SBMA iPSC clones (1E-12, 2E-16, 3E-10, and 4E-5) were used and the data are presented as the average of four clones for the controls and SBMA.

### iPSC culture and differentiation

iPSCs were differentiated into spinal MNs, as previously described [20]. iPSCs were maintained on mitomycin-C-treated SNL murine fibroblast feeder cells in 0.1% gelatin-coated tissue culture dishes in hESC medium and were used for MN induction. For differentiation, hiPSC colonies were detached using a dissociation solution (0.25% trypsin, 100 μg/ml collagenase IV (Gibco), 1 mM CaCl_2_, and 20% KSR) and cultured in suspension in bacteriological dishes in standard hESC medium, after the removal of SNL feeder cells, with incubation for 1–2 h in gelatin-coated dishes. On day 1, the medium was changed to human embryoid body (hEB) medium containing DMEM/F-12, 5% KSR, 2 mM L-glutamine, 1% NEAA, and 0.1 mM 2-ME with 300 nM LDN-193189 (Sigma-Aldrich, USA), 3 μM SB431542 (Tocris, UK), and 3 μM CHIR99021 (FCS, USA). On day 2, the medium was changed to fresh hEB medium containing 300 nM LDN-193189, 3 μM SB431542, and 3 μM CHIR99021, and 1 μM retinoic acid (RA) (Sigma-Aldrich, USA). From day 4 to day 14, hEBs were cultured in hEB medium containing 1 μM RA and 1 μM purmorphamine (Calbiochem, Germany), and the medium was changed every 2–3 days. On day 14, hEBs were enzymatically dissociated into single cells using TrypLE Select (Thermo Fisher Scientific, USA). The dissociated cells were plated on poly-l-ornithine (PO) and recombinant mouse Laminin (Thermo Fisher Scientific, USA), or growth-factor-reduced Matrigel (33 × dilution, thin coated; Corning)-coated dishes at a density of 5 × 10^4^–1 × 10^5^ cells/cm^2^ and cultured in motor neuron medium (MNM) consisting of media hormone mix (MHM) or KBM Neural Stem Cell medium (Kohjin Bio, Japan) [58] supplemented with 2% B27 supplement (Thermo Fisher Scientific, USA), 1% NEAA, 50 nM RA, 500 nM purmorphamine, 10 μM cyclic AMP (cAMP) (Sigma-Aldrich, USA), 10 ng/mL recombinant BDNF (R&D systems, USA), 10 ng/mL recombinant GDNF (R&D systems, USA), 10 ng/mL recombinant human IGF-1 (R&D systems, USA), and 200 ng/mL L-ascorbic acid (Sigma-Aldrich, USA) for up to 4 weeks in 5% O_2_ atmosphere. 10 nM DHT (Sigma-Aldrich, USA) was added to MN culture at the day of lentiviral infection (4 days after monolayer differentiation). Half of the medium was changed every 2–3 days.

### Immunocytochemistry

Cells were fixed in 4% paraformaldehyde for 15–25 min at room temperature. After blocking in blocking buffer (PBS containing 10% FBS and 0.3% Triton X-100), the cells were incubated with primary antibodies overnight at 4 °C. The cells were then rinsed with PBS three times and incubated with Alexa 488-, Alexa 555-, or Alexa 647- conjugated secondary antibodies (Thermo Fisher Scientific) for 1 h at room temperature. Nuclei were stained with 10 μg/ml Hoechst 33258 (Sigma-Aldrich, USA). The cells were then rinsed with PBS three times, mounted on slides, and examined using IX83 (Olympus, Japan). The primary antibodies used in these analyses were listed in Additional file 3: Table S9.

### Teratoma formation assay

Each iPSC clone was harvested in dissociation solution, collected into tubes, and centrifuged, and the resulting pellets were suspended in hESC medium with 10 μΜ Y-27632 (Wako, Japan), which is a Rho-associated coiled-coil forming kinase (ROCK) inhibitor. Then, 1 × 10^5^–5 × 10^5^ cells were injected into the testes of NOD/ SCID mice (Charles River, USA). At 8–10 weeks after injection, the tumors were dissected and fixed with PBS containing 4% PFA. Paraffin-embedded tissue was sliced and stained with hematoxylin and eosin. Images were obtained using a BZ-9000 microscope (Keyence, Japan).

### CAG repeat sizing

DNA was extracted using DNeasy Blood & Tissue kits (Qiagen, Germany). PCR amplification of the CAG repeat in the AR gene was performed using a fluorescent-labeled forward primer (5′-TCCAGAATCTGTTCCAGAGCGTGC-3′) and an unlabeled reverse primer (5′-GCTGTGAAGGTTGCTGTTCCTCAT-3′). Detailed PCR conditions were described previously [58]. For determining the CAG repeat numbers of each PCR product, capillary electrophoresis and direct sequencing were performed using the 3730xl DNA Analyzer (Thermo Fisher Scientific, USA). Fragment analysis was performed using Peak Scanner™ Software v1.0. Sanger sequencing was performed from both 5′ and 3′ sides using a forward sequence primer (5′- TGCGCGAAGTGATCCAGAAC-3′) and a reverse sequence primer (5′- TTGGGGAGAACCATCCTCAC-3′).

### RNA isolation and quantitative RT-PCR analysis

RNA was isolated using a RNeasy mini kit (Qiagen, Germany) and then converted into cDNA using SuperScript III reverse transcriptase (Thermo Fisher Scientific, USA) and Oligo dT primers as described previously [58, 59]. Real-time quantitative RT-PCR were performed as previously described using SYBR Premix ExTaq II and the StepOnePlus or the QuantStudio 7 Real-Time PCR system. The amount of cDNA was normalized to that of human- specific *β-ACTIN* mRNA. The primer sequences and PCR cycling conditions are listed in Additional file 3: Table S10.

### Generation of the *HB9*^*e438*^*::Venus* lentivirus

Lenti-X^TM^ 293T cells cultured in DMEM supplemented with 10% FBS in 150 mm dishes were transfected with 16 μg of pSIN2-*HB9*^*e438*^-βglo-Venus (a variant of yellow fluorescent protein (YFP) with fast and efficient maturation [60]) or pSIN2-βglo-Venus and 10 μg of each of two packaging vectors (pCMV-VSV-G-RSV-Rev and pCAG-HIVgp, kindly provided by Dr. Hiroyuki Miyoshi) with 200 μL of polyethylenimine (Polysciences, Inc., USA), and the medium was changed to the Freestyle 293 expression medium (Thermo Fisher Scientific, USA) the next day. Three days after the medium change, the culture supernatant was harvested and centrifuged at 25,000 rpm for 90 min at 4 °C in an Optima LE-80 K ultracentrifuge (Beckman Coulter, USA). After discarding the supernatant, 80 μL of PBS/150 mm dish was added to the pellet, which was resuspended by repeated pipetting to obtain the *HB9*^*e438*^*::Venus* reporter lentivirus. Lentiviral infection was performed on the day 4 of monolayer MN differentiation. For lentiviral infection, *HB9*^*e438*^*::Venus* in Opti-MEM (Thermo Fisher Scientific, USA) was added to a MN culture, followed by incubation for 2 h, after which the total medium was changed to MNM.

### Flow cytometry

For flow cytometric analysis and cell sorting, iPSC-derived MNs were dissociated 4 weeks after infection with the *HB9*^*e438*^*::Venus* lentivirus using Tissue Dissociation Kits (Miltenyi Biotec) according to the manufacturer’s instructions. The dissociated cells (5 × 10^4^–1 × 10^5^ cells) were suspended in 50–100 μl of Hanks’ balanced salt solution (HBSS) (Thermo Fisher Scientific, USA) containing 2% FBS, 10 mM HEPES, and 1 μg/ml 7-AAD. The cells were then analyzed and sorted based on the expression of the *HB9*^*e438*^*::Venus* reporter using a FACSAria III cell sorter (BD Biosciences, USA).

### RNA sequencing and data analysis

The yield and quality control of total RNA were measured using Nano Drop 2000c (Thermo Fisher scientific, USA) and Agilent RNA6000 Nano Kit (Agilent technologies, USA), respectively. The 2100 Bioanalyzer system (Agilent technologies, USA) was used to qualify RNA integrity number (RIN). RNA samples with sufficient RIN values (more than 9.2) were subjected to the generation of mRNA libraries using Illumina TruSeq protocols for poly-A selection, fragmentation, and adaptor ligation, according to the manufacturer’s instructions (TruSeq RNA Sample Prep Kit v2). Quantification of libraries was performed using Qubit3.0 (Thermo Fisher), Agilent 2200 TapeStaition System (Agilent), and qPCR analysis by Kapa Library Quantification Kit (TakaRa). The multiplexed libraries were sequenced as 75 nt pair end runs on an Illumina NextSeq500 system (San Diego, CA). Sequence reads were mapped to the reference human genome (GRCh38/hg38) using STAR (2-pass mode, version2.7.1a). We then excluded reads mapped to rRNA and tRNA regions. Annotation of the rRNA and tRNA regions were obtained from the UCSC Table Browser. Read counts of transcripts (feature counts [61]) were calculated using FeatureCounts of the Rsubread package. Ensembl gene annotation (Homo_sapiens.GRCh38.96.chr.gtf) was used for the transcript counts. DEGs based on the Wald test were analyzed using DESeq2 [62]. The expression data were grouped using a hierarchical clustering algorithm in Cluster 3.0 software (http://bonsai.hgc.jp/~mdehoon/software/cluster/software.htm) [63] by average linkage with the Euclidean distance, and visualized by Java TreeView software (http://jtreeview.sourceforge.net/) [64].

### Gene set enrichment analysis (GSEA)

GSEA is a computational method that determines if a priori defined set of genes show statistically significant, concordant differences between two biological states [23], and is accessible at http://www.broadinstitute.org/gsea/index.jsp. The expression profile data were analyzed using GSEA 4.0.1. The gene sets were downloaded from the Molecular Signatures Database (MSigDB) and C2 (curated gene sets: chemical and genetic perturbation (CGP) and Canonical pathway (CP)) and C5 (Gene ontology gene sets) were used for the GSEA.

### Statistical analysis

For the statistical analysis in the quantitative RT-PCR, either the Student’s t-test or the Welch’s t-test was used. For the differential analysis of mRNAs between control and SBMA MNs using DESeq2, adjusted *p*-value for a false discovery rate (FDR) correction was performed by the Benjamini–Hochberg (B-H) method. The gene set enrichment analysis was performed using the Fisher’s exact test and corrected with the B-H FDR.

## Supplementary information

**Additional file 1: Figure S1.** Evaluation of established iPSCs. Related to Fig. 1. (A) Immunocytochemical analysis of the established iPSC clones for pluripotent markers OCT4 and NANOG. Scale bar, 100 μm. (B) Hematoxylin and eosin staining of teratomas derived from the established iPSC clones. Scale bar, 200 μm.

**Additional file 2: Figure S2.** Karyotype analysis of the established iPSC clones via G-banding analysis. Related to Fig. 2. All clones showed normal karyotypes, 46, XY.

**Additional file 3: Table S1.** DEGs in heatmap (NT). Related to Fig. 5C. **Table S2.** DEGs in heatmap (HPF). Related to Fig. 5D. **Table S3.** Profiles of GSEA. **Table S4.** Gene ontology terms (down in NT). List of GO terms negatively enriched in SBMA-MNs of NT (FDR < 0.2). **Table S5.** Gene ontology terms (down in HPF). List of GO terms negatively enriched in SBMA-MNs of HPF (FDR < 0.01). **Table S6.** Pathway terms (down in NT). List of pathway terms negatively enriched in SBMA-MNs of NT (FDR < 0.01). **Table S7.** Pathway terms (down in HPF). List of pathway terms negatively enriched in SBMA-MNs of HPF (FDR < 0.01). **Table S8.** The extracted upregulated genes in SBMA-MNs in GSEA. List of 96 genes that were significantly upregulated in enriched GO and pathway terms with *p* < 0.05 and significantly higher (> 2-fold) expression levels in SBMA-MNs than in control-MNs. **Table S9.** Primer sequences and cycling conditions for quantitative RT-PCR. **Table S10.** Antibodies for immunocytochemistry.

## Data Availability

The datasets supporting the conclusions of this article are available in the following repositories; RNA sequence expression data in GEO. [http://www.ncbi.nlm.nih.gov/geo/]. (accession number: GSE142612)

## Abbreviations

AChR: Acetylcholine receptor
ALS: Amyotrophic lateral sclerosis
AR: Androgen receptor
*CALCA*: *Calcitonin-related polypeptide α*
*CXCL14*: *CXC motif chemokine ligand 14*
DHT: Dihydrotestosterone
DM1: Myotonic dystrophy
EB: Embryoid body
*FAM135B*: *Family with sequence similarity 135 member B*
FDR: False discovery rate
GO: Gene ontology
GPCR: G protein-coupled receptor
*GRID2*: *Glutamate ionotropic receptor delta type subunit 2*
*GRIK1*: *Glutamate ionotropic receptor kainate type subunit 1*
*GRM2*: *Glutamate metabotropic receptor 2*
GSEA: Gene set enrichment analysis
HD: Huntington disease
hESC: Human embryonic stem cell
hiPSC: Human induced pluripotent stem cell
HPF: High positive fraction, purified motor neurons
*IGF-1*: *Insulin-like growth factor-1*
iPSC: Induced pluripotent stem cell
Lgr5: Leucine-rich repeat-containing G-protein coupled receptor 5
*MFRP*: *Membrane-type frizzled-related protein*
MN: Motor neuron
NES: Normalized enrichment score
NF: Negative fraction
NMJ: Neuromuscular junction
NT: No treatment, unpurified motor neurons
*NTF3*, NT-3: *Neurotrophin-3*
*RGS4*: *Regulator of G protein signaling 4*
*RGS5*: *Regulator of G protein signaling 5*
*RSPO2*: *R-spondin 2*
SBMA: Spinal bulbar muscular atrophy
*SYT6*: *Synaptotagmin6*
*SYT9*: *Synaptotagmin9*
*WNT2B*: *Wnt family member 2B*
*WNT3A*: *Wnt family member 3A*
