# Supplementary figures and images for: Unveiling synapse pathology in spinal bulbar muscular atrophy by genome-wide transcriptome analysis of purified motor neurons derived from disease specific iPSCs
Source: Mol Brain. 2020 Feb 19;13:18. doi: 10.1186/s13041-020-0561-1 (PMC7029484; doi:10.1186/s13041-020-0561-1)

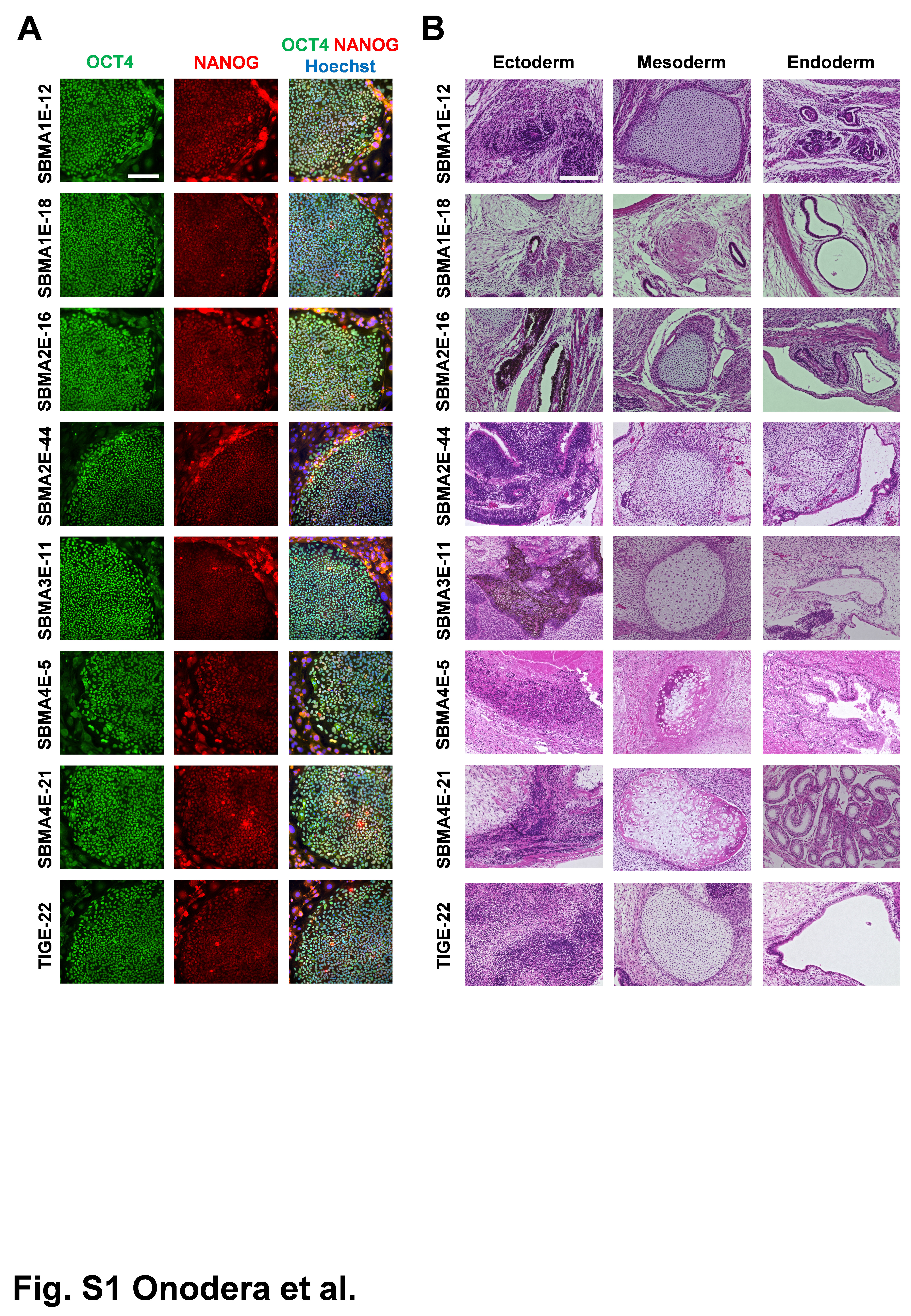

Supplement: Supplementary file 1 — Additional file 1: Figure S1. Evaluation of established iPSCs. Related to Fig. 1. (A) Immunocytochemical analysis of the established iPSC clones for pluripotent markers OCT4 and NANOG. Scale bar, 100 μm. (B) Hematoxylin and eosin staining of teratomas derived from the established iPSC clones. Scale bar, 200 μm. [file 13041_2020_561_MOESM1_ESM.jpg]

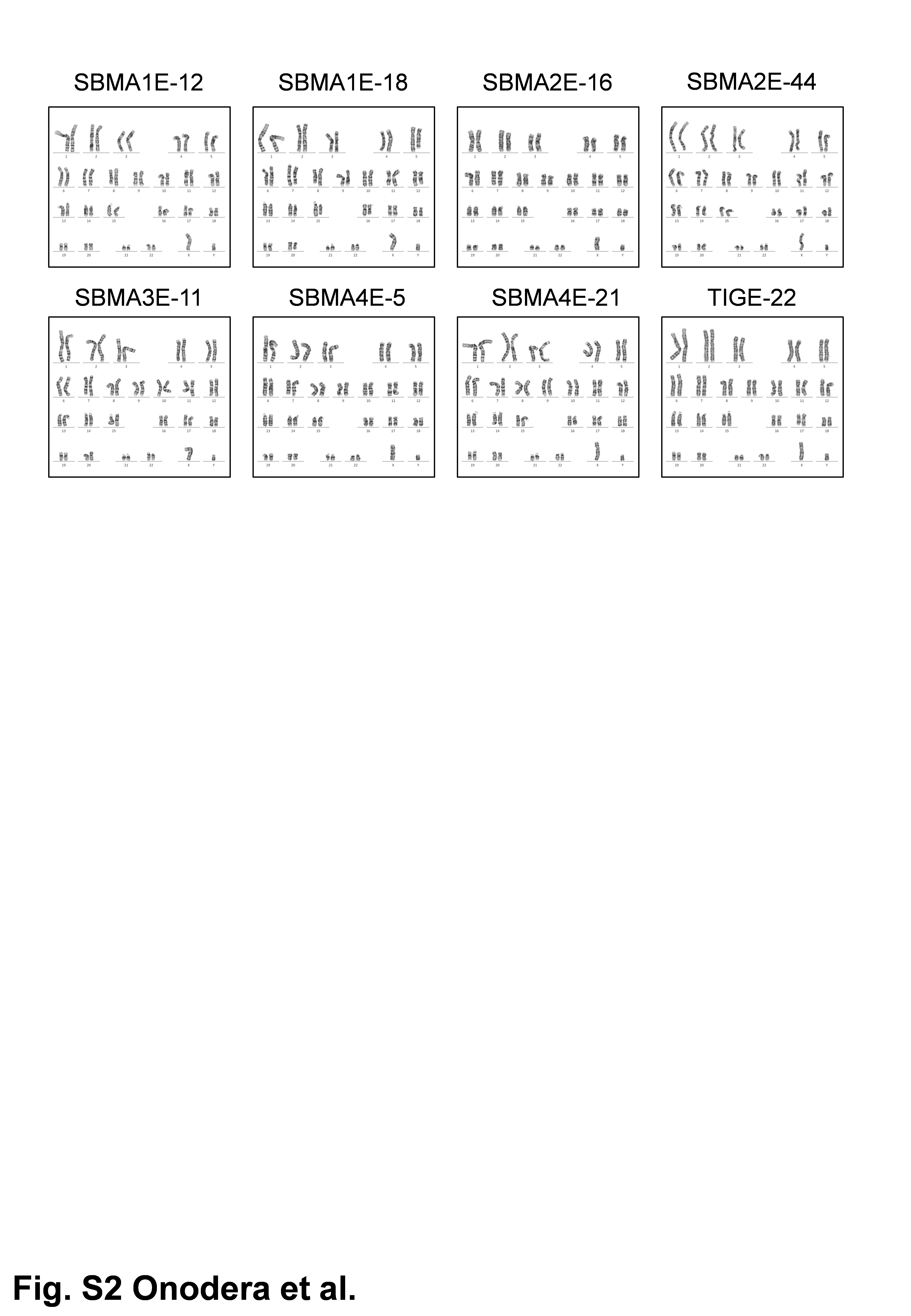

Supplement: Supplementary file 2 — Additional file 2: Figure S2. Karyotype analysis of the established iPSC clones via G-banding analysis. Related to Fig. 2. All clones showed normal karyotypes, 46, XY. [file 13041_2020_561_MOESM2_ESM.jpg]
